# Changes in severity of traffic related trauma aged 18+ admitted to a local level 2 trauma centre after the introduction of the Mobile Emergency Care Unit at Southern Funen. A pilot study

**DOI:** 10.1186/1757-7241-23-S1-A5

**Published:** 2015-07-16

**Authors:** Stine Hebsgaard, Stine T Zwisler, Jens Lauritsen

**Affiliations:** 1 Department of Anaesthesiology & Intensive Care, OUH Odense University Hospital, Svendborg, Denmark; 2 Accident Analysis Group, Department of Orthopaedics, OUH Odense University Hospital, Odense, Denmark

## Background

Centralizing and specializing in Danish health care is ongoing. Around the country Mobile Emergency Care Units (MECU) are introduced during the past years. Pre-hospital triage is important to evaluate at which trauma level the severely injured patients must be received. The aim of our study was to evaluate changes in the severity in 18+ traffic related trauma admitted to a level 2 centre at Odense University Hospital, Svendborg Hospital (OUH, SH) after the introduction of a local MECU.

## Methods

The study was a retrospective study covering a ten-year period from 2002-2012. All admissions from traffic accidents to OUH, SH were extracted from the hospital inpatient registry for patients aged 18+. Based on clinical record reviews and radiology findings, we decided if the patient was multi trauma (MT) defined as received by trauma response team and/or CT trauma scanned. We evaluated the diagnoses and assigned whether maximum Abbreviated Injury Score (mAIS) was three or more (severe injury). A total of 363 traffic injury patients were identified. Five were undeterminable in MT status and 137 non-MT patients were excluded, giving 221 adult MT cases for analysis. From the years 2002-2006 (118 MT) - before and 2010-2012 (46 MT) - after implementing the MECU 24/7 in Svendborg September 1^st ^2009. The years 2007-2009 were extracted since the MECU was launched part time in 2007. The study was performed as a pilot study including patients born on the 1st to 6th of each month.

## Results

Proportion of mAIS ≥ 3 in the years before implementing the MECU in Svendborg was 17.1% (CI: 10.2-24.0) versus 23.9% (CI: 11.1-36.7) in the years after the implementation (p = 0.32).

## Conclusions

There was no significant change in the proportion of severely injured patients admitted to this level 2 trauma centre after implementation of the local MECU in this study.

